# A hierarchical multi-agent reinforcement learning framework with high-level guidance from large language models

**DOI:** 10.1038/s41598-026-54971-6

**Published:** 2026-05-25

**Authors:** Jinyin Bai, Wei Zhu, Xiangchen Wang, KaiYang Kou, Shiluo Guo, Shuhong Liu, Dong Li, Tianjin Ni, Jinji Zhou, Yihao Zhong

**Affiliations:** 1 National University of Defense Technology, Changsha, 410000 China; 2 Information Support Force Engineering University, Wuhan, 430000 China; 3 Beijing Satellite Navigation Center, Beijing, 100000 China

**Keywords:** Multi-agent reinforcement learning, Large language models, Hierarchical reinforcement learning, Semantic reward shaping, Dynamic action masking., Engineering, Mathematics and computing

## Abstract

Multi-agent reinforcement learning (MARL) has achieved substantial progress in cooperative decision-making, but learning remains difficult in environments with sparse rewards, long decision horizons, and strong inter-agent coupling. Existing methods usually optimize low-level policies directly from numerical observations, which can limit sample efficiency and make it difficult to incorporate structured strategic guidance. Here we propose LEHCA, a hierarchical MARL framework that uses a large language model as a coarse-timescale Commander to provide high-level semantic guidance for value-decomposition-based policy learning. The Commander receives structured textual summaries derived from observable environment information and generates strategic sub-goals, semantic reward-shaping rules, and action-level constraints. These outputs are grounded in low-level QMIX-based agents through two modular mechanisms: semantic reward shaping, which converts abstract sub-goals into dense auxiliary learning signals, and dynamic action masking, which guides exploration toward strategically relevant actions. Experiments on eight StarCraft multi-agent challenge scenarios show that LEHCA improves over QMIX across the evaluated maps in the reported metrics, with larger gains in heterogeneous, sparse-reward, and outnumbered settings. Additional comparisons with QPLEX, MAVEN, and MAPPO on representative scenarios indicate stronger early-stage learning efficiency, while ablation studies and non-LLM control variants show that both hierarchical guidance and LLM-generated semantic reasoning contribute to performance. A lightweight cooperative navigation experiment in the multi-agent particle environment further suggests that the framework can be instantiated beyond StarCraft micromanagement. These results support hierarchical LLM-guided MARL as a promising approach for improving learning efficiency, coordination, and interpretability in cooperative multi-agent systems.

## Introduction

As the scale of complex systems continues to grow and environmental dynamics become increasingly uncertain, enabling efficient coordination and robust decision-making among multiple agents under decentralized settings has become a central challenge in artificial intelligence. Multi-agent reinforcement learning (MARL) provides a general framework in which multiple agents learn cooperative or competitive behaviors through interactions in a shared environment, and has been applied to domains such as game playing, multi-robot coordination, intelligent transportation, and distributed resource management^1–3^. Recent advances in deep reinforcement learning have further improved the performance of MARL in simulated environments.

Despite these advances, existing MARL methods still face key practical limitations when applied to complex environments characterized by long decision horizons, sparse and delayed rewards, and strong inter-agent coupling. In such settings, long-term credit assignment and effective exploration remain challenging, and the non-stationarity induced by concurrently learning agents often leads to unstable or slow convergence. Moreover, most MARL approaches rely on end-to-end numerical function approximation, which makes it difficult to explicitly represent high-level task structures or strategic intent. As a result, learning efficiency and interpretability remain limited in many coordination-intensive settings, as reflected in recent applied intelligent-system studies^4^. More generally, related planning and decision difficulties also arise in broader autonomous systems, including unmanned vehicle navigation in unstructured off-road environments^5^.

To address these challenges, a variety of MARL techniques have been proposed, including value decomposition methods such as VDN^6^, QMIX^7^, and QTRAN^8^, as well as actor–critic approaches such as MADDPG^9^ and MAPPO^10^. While these methods alleviate coordination conflicts and non-stationarity to some extent, they primarily operate at the level of low-level numerical optimization and remain limited in their ability to support long-horizon strategic planning, complex tactical reasoning, and generalization beyond the training distribution.

The recent rapid development of large language models (LLMs) offers a promising new perspective for overcoming these limitations. Owing to large-scale pretraining, LLMs exhibit strong capabilities in knowledge integration, logical reasoning, and long-horizon task planning^11^, and can decompose complex objectives into structured intermediate subgoals through explicit reasoning-oriented prompting^12,13^. These properties closely align with the requirements of multi-agent systems for high-level strategic cognition, stage-wise goal specification, and interpretable decision-making. Furthermore, the use of natural language as a unified representation enables LLMs to incorporate human expert knowledge and symbolic priors, providing new opportunities for high-level decision support in embodied and interactive systems^14,15^.

More broadly, it is helpful to connect our setting to multimodal control and prompting. Representative studies include multimodal reinforcement learning for human–robot collaboration, sound-guided multimodal representation learning and exploration, and vision–language prompting for robot manipulation^16–18^. Much of this research develops end-to-end multimodal sensorimotor policies, cross-modal representation learners, or prompting pipelines in which heterogeneous sensing and language jointly condition low-level actions. LEHCA is not designed in this mold: we do not learn a single policy that directly maps raw multimodal streams to primitive actions. Instead, LEHCA adopts a hybrid numerical–textual interface to the same observable environment state—numerical features for decentralized MARL execution and a structured natural-language summary for high-level reasoning by the LLM Commander—thereby separating semantic planning from tactical learning while leaving the MARL agents’ conventional action interface unchanged.

Recent studies have begun to integrate LLMs into reinforcement learning and multi-agent systems as high-level planners or reward generators. However, existing approaches still suffer from several limitations. First, LLMs are often treated as external or one-shot tools^19^, leading to weak coupling with the underlying MARL learning process and limited adaptability in dynamic environments. Second, there is a significant mismatch between the long temporal scale of LLM reasoning and the high-frequency decision-making required by MARL agents^20^. Third, LLM-generated strategies may be semantically plausible but infeasible under environmental constraints, and without systematic constraints and feedback mechanisms, such outputs may even degrade decision reliability^21^. Consequently, how to achieve deep coordination between LLMs and MARL across cognitive hierarchies, temporal scales, and learning mechanisms remains an open problem.

To address these issues, this paper proposes an LLM-empowered hierarchical cognitive architecture (LEHCA) for multi-agent reinforcement learning. Inspired by the human strategic–tactical decision-making paradigm, LEHCA introduces a clear functional decomposition and well-defined information interfaces. In this architecture, the LLM serves as a high-level strategic cognition and expert reasoning module, responsible for global situation understanding, long-horizon goal planning, task decomposition, and semantic reward generation, conditioning on structured textual summaries of observable information rather than on raw simulator tensors or training logs. The lower-level MARL agents act as tactical executors, learning fine-grained action policies through high-frequency interactions with the environment under structured goals and constraints provided by the LLM; tactical learning retains a CTDE-style QMIX backbone (centralized training, decentralized execution). Unlike existing approaches that employ LLMs as heuristic add-ons, LEHCA tightly couples LLM-generated cognitive outputs with the reward mechanism and policy optimization process, ensuring stable and controllable propagation of high-level strategic intent.

The main contributions of this work are summarized as follows:

- We propose a hierarchical multi-agent reinforcement learning framework that integrates high-level guidance from a large language model with value-decomposition-based learning. The tactical layer retains a CTDE-style QMIX backbone (centralized training with decentralized execution for the learned policies), while semantic guidance from the Commander operates at a coarser temporal scale on structured textual summaries of observable information.
- We introduce an LLM-driven semantic reward shaping mechanism and a task-guided policy learning scheme with dynamic action masking to facilitate structured exploration and improve learning efficiency in sparse-reward environments.
- We report experiments on eight SMAC^22^ scenarios, together with additional comparisons on representative maps against QPLEX, MAVEN, and MAPPO, which show improvements over QMIX across the evaluated SMAC settings in our reported metrics and competitive outcomes against stronger baselines on the representative subset, with the clearest relative advantages in $\textrm{AUC}_{\textrm{early}}$ and harder coordination regimes.

The remainder of this paper is organized as follows. Section "Related work" reviews related work. Section "Hierarchical cognitive architecture" describes the proposed LEHCA framework and its key components. Section "Experiments" presents experimental results and analysis. Section "Conclusion" concludes the paper and discusses future research directions.

## Related work

### Cooperative MARL under CTDE

Cooperative multi-agent reinforcement learning (MARL) is commonly formulated as a decentralized partially observable Markov decision process (Dec-POMDP), where agents act from local observations while optimizing a shared long-term return^23–25^. Under the centralized training and decentralized execution (CTDE) paradigm, value-factorization methods such as VDN, QMIX, QTRAN, and QPLEX^26^ improve scalability by constraining or transforming the joint action-value function so that decentralized greedy policies remain tractable. Actor–critic methods, including MADDPG and MAPPO, instead employ centralized critics to reduce non-stationarity and have become strong general-purpose baselines in cooperative benchmarks. These approaches substantially advance cooperative MARL, yet they still rely predominantly on low-level numerical optimization and do not explicitly model high-level semantic task structure.

### Hierarchical and exploration-oriented MARL

To address long-horizon credit assignment and inefficient exploration, several MARL methods introduce higher-level structure. MAVEN augments value-based MARL with a shared latent variable to induce temporally extended exploration, while role-based methods such as RODE decompose multi-agent tasks into a higher-level role-selection process and lower-level action execution^27,28^. These studies show that higher-level abstractions can improve coordination and sample efficiency. However, their high-level variables are learned only from interaction data and are not grounded in explicit semantic reasoning or external knowledge. LEHCA is complementary to these methods: it retains a CTDE-style low-level learner through the QMIX backbone but uses an LLM to generate interpretable sub-goals, reward rules, and action constraints that can be updated online.

### Multimodal control learning and multimodal prompting

A related line of work studies how heterogeneous modalities can improve control^29–31^. In human–robot collaboration, multimodal reinforcement learning has been used to learn interaction policies from language and physical-action signals. Auxiliary modalities such as sound can improve representation quality and exploration. VIMA illustrates how robot manipulation can be specified through multimodal prompts that interleave textual and visual tokens. Collectively, these lines of work suggest that richer cross-modal representations can benefit control and prompting. Nevertheless, they are largely oriented toward single-agent or robotic platforms and often emphasize fused end-to-end controllers or prompting pipelines. By contrast, LEHCA targets cooperative MARL under decentralized execution, using semantic guidance to structure coordination among multiple learners while retaining CTDE-style centralized training and decentralized tactical execution through the QMIX backbone, rather than replacing cooperative decentralized MARL with a single multimodal motor policy.

### LLM-guided decision making

Large language models have recently been used as planners, policy priors, reward designers, and interactive decision modules in sequential tasks^32–34^. Existing studies suggest that LLMs can provide useful task decompositions, long-horizon plans, and interpretable guidance for embodied or interactive agents. However, many existing approaches use LLMs as static priors, one-shot planners, or loosely coupled external tools, which limits their adaptability during policy learning. LEHCA differs in that the LLM is embedded inside the learning loop and affects both reward construction and policy search through dynamic action masking. This tighter coupling is designed to better align high-level reasoning with decentralized multi-agent learning.

The LLM-driven high-level guidance in the LEHCA framework can be interpreted as a form of *biased exploration* that incorporates a strong semantic prior. In typical cooperative multi-agent exploration methods, such as cooperative multi-agent exploration and subspace-aware exploration, the central idea is to encourage agents to collaboratively visit unknown or information-rich regions of the state space through intrinsic rewards or structured exploration strategies. However, the exploration bias introduced by these methods largely originates from statistical information acquired during learning or from task-agnostic heuristics. In contrast, LEHCA employs an LLM Commander to inject prior knowledge—composed of domain knowledge, tactical common sense, and logical reasoning—into the learning process in the form of sub-goals and action constraints. This mechanism can be regarded as *knowledge-driven exploration*: rather than expanding exploration by increasing randomness or pursuing state-space novelty, it compresses and reshapes the search space, steering it toward regions that are both highly strategically relevant and semantically plausible. Consequently, this approach is complementary to existing exploration techniques: the low-level policy can continue to leverage intrinsic reward-based exploration methods to handle local uncertainty, but only within the strategically feasible region delineated by the LLM. This perspective positions LEHCA as a novel pathway for addressing the multi-agent exploration challenge under sparse rewards at the semantic level.

In summary, existing MARL methods provide strong low-level optimization, hierarchical MARL methods provide abstract structure, and multimodal/LLM-based approaches provide semantic reasoning. The central gap addressed by LEHCA is how to combine these advantages within a single hierarchical framework that preserves CTDE-style tactical learning through a QMIX backbone while enabling interpretable, adaptive, and strategically grounded high-level guidance.

## Hierarchical cognitive architecture

This section presents the proposed LLM-empowered hierarchical cognitive architecture (LEHCA) for multi-agent reinforcement learning. We first introduce the overall design philosophy and cognitive decomposition, followed by a description of the high-level strategic cognition mechanism, semantic reward shaping, and task-guided multi-agent policy learning.

### Overall architecture

In complex multi-agent environments, conventional end-to-end MARL methods are required to simultaneously address long-horizon strategic planning, real-time tactical decision-making, and long-term credit assignment. Learning all these responsibilities within a single numerical model significantly increases training difficulty and often results in limited interpretability and generalization.

**Fig. 1:**
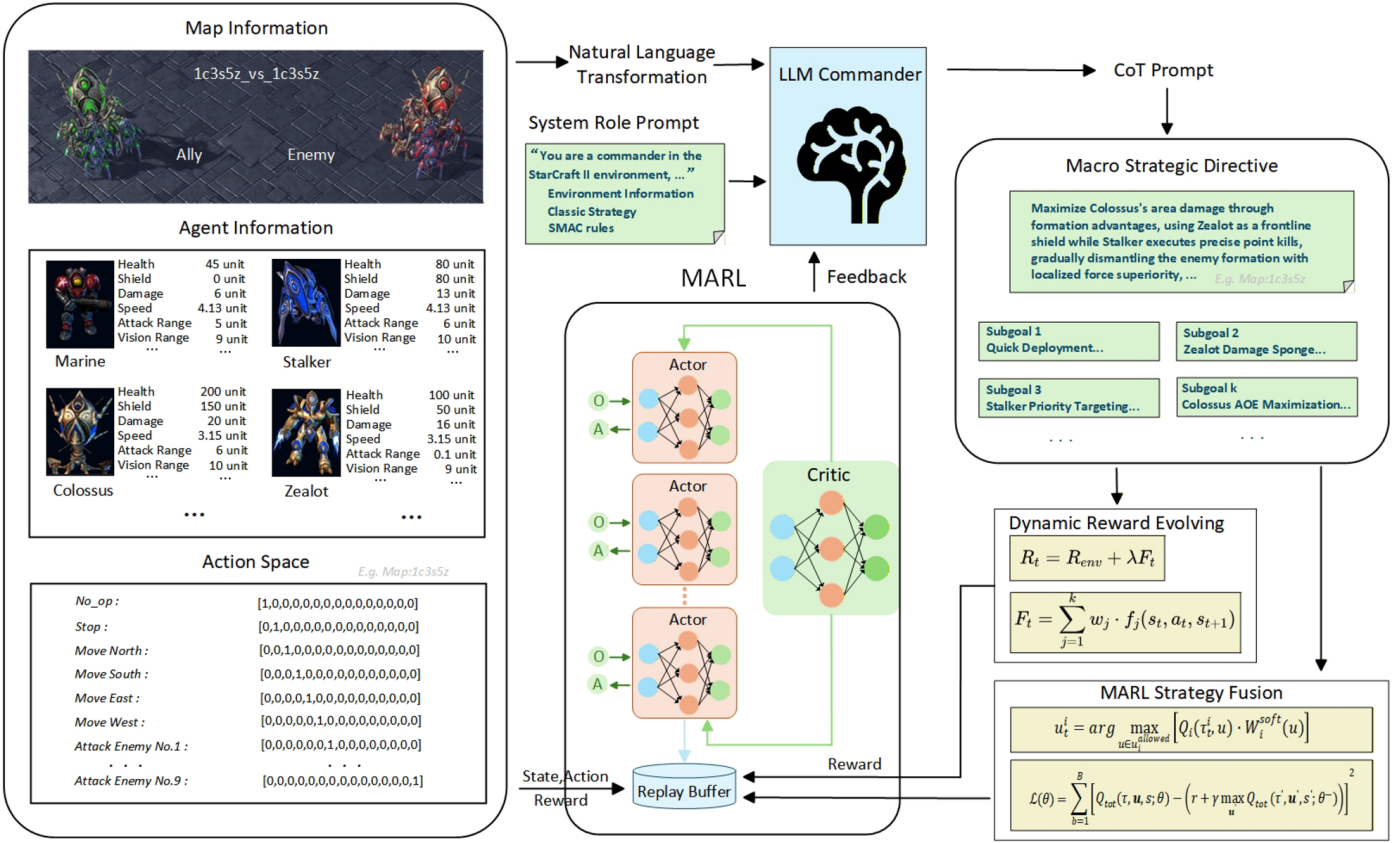
LEHCA for multi-agent reinforcement learning. Observable information from agents is transformed into structured semantic descriptions $d_t$ and fed into the LLM commander. Through structured reasoning prompts, the LLM generates high-level strategic decisions, sub-task objectives, and semantic rewards, and guides the policy learning and execution of the underlying MARL agents via a dynamic action masking mechanism.

Inspired by the human strategic–tactical decision-making paradigm, LEHCA explicitly decomposes the decision process into distinct cognitive layers operating at different temporal and abstraction scales. As illustrated in Fig. 1, the proposed architecture consists of two complementary components: a high-level strategic cognition layer and a low-level tactical execution layer, which interact through well-defined semantic and numerical interfaces to form a closed-loop decision system.

The high-level strategic cognition layer is implemented by a large language model, referred to as the LLM Commander, which serves as an expert-level reasoning and planning module. Its main responsibilities include: (i) global situation understanding and complex context analysis based on structured *semantic summaries* of observable information (not raw simulator tensors); (ii) formulation of team-level strategic objectives and long-horizon planning; (iii) dynamic decomposition of strategic goals into executable sub-tasks with assigned priorities; (iv) generation of semantic reward signals aligned with strategic intent; and (v) specification of behavioral norms and action constraints to regulate tactical execution.

The low-level tactical execution layer consists of multiple MARL agents that interact directly with the environment at a high frequency. Each agent makes decentralized decisions based on local observations and learns fine-grained action policies under the guidance of sub-task goals, auxiliary rewards, and action constraints provided by the LLM Commander. Standard MARL algorithms are employed to optimize tactical policies and achieve locally optimal execution of assigned sub-tasks.

Regarding information flow, agents’ local observations $\{o_i^t\}_{i=1}^{N}$ (together with any interface-level aggregation that does not exceed the observable information used for decentralized MARL) are passed through a semantic transformation module that produces a structured natural-language bundle $d_t$ suitable for LLM reasoning. The LLM Commander conditions only on $d_t$ (and the prompt template), not on privileged environment-only tensors or training statistics such as rolling win rates. Through prompt engineering, the role, inputs, and outputs of the LLM Commander are explicitly defined. Inspired by structured reasoning, acting, and feedback-aware prompting paradigms, the Commander is prompted to produce concise rationale-style summaries together with structured strategic decisions and corresponding sub-task objectives. Meanwhile, the LLM dynamically generates and refines goal-oriented semantic rewards. The resulting sub-task goals and action constraints are incorporated into the MARL optimization process via a dynamic action masking mechanism, thereby guiding exploration and policy learning at the numerical level.

The key advantage of this hierarchical cognitive design lies in decomposing complex decision-making into high-level strategic reasoning and low-level tactical learning. The LLM focuses on long-horizon planning, expert reasoning, and knowledge-driven guidance, while MARL agents handle fine-grained control through high-frequency interaction. This separation effectively mitigates the limitations of conventional MARL in long-term dependency modeling, reward sparsity, and decision interpretability.

To balance decision quality and computational efficiency, LEHCA adopts a hierarchical multi-timescale design. In the implementation reported here, the LLM Commander is invoked on a *fixed schedule* every $F_{\textrm{update}}$ environment steps; the semantic summary $d_t$ is rebuilt on the same schedule so that Commander invocation frequency and semantic refresh frequency stay aligned. Event-triggered Commander calls (e.g., on large state changes or sub-task completion) are a natural extension for adaptive deployments but are not used in the reported empirical evaluation. This schedule amortizes LLM cost while keeping strategic guidance synchronized with the textual interface.

### Environment interaction and semantic representation

The environment interaction module provides the representational interface that connects low-level numerical control with high-level semantic reasoning. In LEHCA, the environment is represented through two coupled views. The low-level MARL agents consume standard local observations for decentralized execution, while centralized training follows the same CTDE conventions as the underlying QMIX implementation (including access to the global state for the mixing network during training). In parallel, a structured natural-language summary $d_t$ is built from the same *observable* interface (e.g., $\{o_i^t\}$ and non-privileged summaries exposed by the interface builder) and is the sole situational input to the LLM Commander. These two views should not be interpreted as independent sensors; rather, they constitute a hybrid numerical–textual representation grounded in compatible information sources. This design allows the strategic layer to exploit semantic abstraction without feeding the LLM privileged ground-truth fields that the decentralized policies cannot access at execution time.

#### Structured natural language representation

The objective of the environment interaction module is to map high-dimensional, heterogeneous, and dynamically changing observations into structured natural language representations that are suitable for LLM reasoning while preserving key tactical features. This process consists of the following steps:

- *Data filtering and aggregation* Irrelevant or redundant information is filtered out, and related observations are semantically aggregated, such as summarizing multiple allied units into an overview of friendly force status and enemy units into an enemy force profile.
- *Key entity identification* Strategically significant entities are identified, including high-threat enemy units, high-value targets, vulnerable friendly units, and critical terrain or strategic positions.
- *Relation inference and situation description* Tactical relationships are inferred based on spatial, categorical, and attribute information, such as attack relations, encirclement situations, focus-fire priorities, and potential risk areas.
- *Template-based semantic generation* Predefined language templates and rule-based mappings are employed to generate concise, consistent, and semantically precise textual descriptions. Templates can be adapted according to different combat phases or tactical contexts.

This semantic representation process is executed whenever the Commander is scheduled to refresh (every $F_{\textrm{update}}$ steps in our implementation), producing the textual bundle $d_t$ passed to the Commander update in Algorithm 1. Between refreshes, the latest $d_t$ and parsed Commander outputs remain fixed until the next rebuild. The structured representation reduces input dimensionality for the LLM and improves reasoning efficiency while minimizing semantic ambiguity.

#### Prompt engineering and strategic constraints

To ensure that the LLM Commander produces coherent, executable, and learning-oriented strategic guidance, a systematic prompt engineering strategy is adopted to constrain and guide its reasoning behavior^35^. The prompt template includes the following components:

- *Role and capability specification* The LLM is explicitly assigned the role of a domain-expert commander responsible for strategic analysis and guidance rather than direct low-level control.
- *Task objective definition* The overall objective and success criteria for the current phase are clearly stated.
- *Structured environment input* The semantic bundle $d_t$ produced by $\mathrm {Semantic\_Transform}(\cdot )$, including allied and enemy status, map information, and temporal context, is provided in a structured textual format.
- *Behavioral constraints and preferences* Strategic-level hard constraints and soft preferences are specified to avoid infeasible or risky decisions and to bias planning toward desired tactical styles.
- *Output format specification* The LLM is required to output decisions in a structured JSON format, facilitating automated parsing by downstream modules such as reward shaping and action masking.

Through this prompt design, the LLM’s reasoning space is constrained to a controllable and task-consistent domain, significantly enhancing the stability and interpretability of high-level decisions.

### High-level strategy generation and task decomposition

High-level strategy generation is a core component of strategic cognition in the LEHCA framework. To improve reliability and reduce unstructured outputs, we use structured reasoning prompts inspired by chain-of-thought prompting to guide the LLM through situation analysis, strategic planning, and task decomposition.

Under this mechanism, the LLM first performs multi-step situation assessment based on structured environmental descriptions, identifying threats, opportunities, and causal relations. It then produces a logically coherent high-level strategy through explicit reasoning steps and decomposes it into a set of concrete, evaluable, and time-bounded sub-task objectives. Each sub-task is assigned a priority reflecting its importance and urgency within the overall strategy.

The dynamically generated sub-task objectives and priorities serve as key inputs for subsequent semantic reward shaping and policy constraint mechanisms, ensuring that strategic intent is accurately propagated into low-level numerical learning. Moreover, the LLM can periodically reassess and adjust the sub-task sequence in response to environmental changes, enhancing adaptability in complex dynamic settings.

Importantly, within the LEHCA framework, the LLM does not access privileged information such as future states, environment rules, or true reward functions. All inputs are derived from the same observable information available to MARL agents and are transformed into structured natural language descriptions. Thus, the LLM’s strategic reasoning constitutes a high-level semantic abstraction rather than information leakage.

### Dynamic semantic reward shaping

To alleviate reward sparsity and inefficient exploration in multi-agent reinforcement learning, LEHCA introduces an LLM-based dynamic semantic reward shaping mechanism that transforms high-level tactical sub-goals generated by the LLM into dense, interpretable, and dynamically adjustable reward signals. This mechanism guides low-level MARL agents toward purposeful exploration while preserving the original task objective.

Under a given macro-strategy, the LLM generates a set of sub-goals $G = \{g_1, g_2, \ldots , g_K\}$, where each sub-goal $g_j$ represents an expected behavior pattern or state transition that agents should achieve during the current strategic phase. Unlike manually designed static rewards, the LLM leverages its semantic reasoning capability to produce natural-language reward rule descriptions for each sub-goal, explicitly specifying reward conditions, magnitudes, and contextual constraints.

For example, for the sub-goal “prioritize eliminating enemy damage dealers”, the LLM may generate the following rule: “Provide a positive team reward for each eliminated enemy ranged unit, and grant an additional guiding reward if the unit is focus-fired within a short time window.”

These natural-language reward rules are processed by a semantic grounding module and converted into computable reward predicates and executable shaping functions $f_j(s_t, a_t, s_{t+1})$, where $s_t$ denotes the training-time state representation used by the MARL learner, $a_t$ the joint action, and $s_{t+1}$ the next state. The LLM itself does not directly observe the true reward function or privileged simulator internals; it only produces rule descriptions and priority weights from the structured semantic bundle $d_t$. The grounding module then maps these descriptions to measurable events or predicates available in the training interface, such as unit elimination, relative distance, focus-fire consistency, or ally protection.

At time step *t*, the shaping reward $F_t$ is defined as a weighted sum of sub-goal rewards:

1 $$\begin{aligned} F_t = \sum _{j=1}^{K} w_j \cdot f_j(s_t, a_t, s_{t+1}) \end{aligned}$$

where $w_j \in [0,1]$ represents the priority weight of sub-goal $g_j$, dynamically assigned by the LLM according to the current strategic context and task urgency.

The total reward received by MARL agents is then given by:

2 $$\begin{aligned} R_t = R_t^{\textrm{env}} + \lambda _t F_t \end{aligned}$$

where $R_t^{\textrm{env}}$ denotes the original environment reward (typically sparse in SMAC), and $\lambda _t \in [0,1]$ controls the relative influence of semantic shaping rewards.

To prevent long-term bias toward shaped rewards, a progressive decay schedule is applied to $\lambda _t$ during training. Larger values are used in early stages to encourage guided exploration, while $\lambda _t$ is gradually reduced as policies mature, ensuring that final optimization primarily depends on the original environment reward.

This dynamic semantic reward shaping mechanism offers several practical advantages: (i) it mitigates reward sparsity by providing dense, goal-oriented feedback; (ii) it aligns exploration with high-level strategic intent through dynamic sub-goal activation and weighting; (iii) it enhances interpretability by expressing reward logic in natural language; and (iv) it reduces the risk of long-term over-dependence on shaped rewards through controlled reward decay. The quality of this shaping mechanism depends on the fidelity of the semantic grounding rules and the consistency of the high-level sub-goals. Therefore, semantic reward shaping should be understood as a structured guidance mechanism rather than a guarantee of optimal reward design.

### MARL policy learning

In the LEHCA framework, the macro-level strategies and dynamic reward shaping signals generated by the LLM Commander are ultimately grounded through the value function learning mechanism of low-level MARL agents. Overall, LEHCA is hierarchical: high-level semantic guidance is produced at a coarse timescale from structured summaries $d_t$, whereas tactical control is learned by an off-policy QMIX stack that retains CTDE-style centralized training and decentralized execution for the underlying low-level policies. We emphasize that the full LEHCA system is not intended to be interpreted as a purely standard CTDE algorithm, because the Commander provides additional coarse-timescale guidance through rewards and masks. In the reported evaluations, the Commander is refreshed using the fixed interval $F_{\textrm{update}}$, and tactical agents select actions using masked greedy or masked *\varepsilon*-greedy scores derived from the latest available Commander guidance. This section presents the value-decomposition-based MARL learning algorithm adopted in this work and focuses on how LLM-generated task objectives and action constraints are integrated into Q-value learning and action selection via a dynamic masking mechanism, enabling task-guided value function learning.

#### MARL algorithm

This work adopts QMIX as the underlying MARL learning algorithm. QMIX is a representative value decomposition method that follows the centralized training and decentralized execution (CTDE) paradigm for the learned tactical policies, making it particularly suitable for cooperative multi-agent tasks. Its core idea is to learn a joint action-value function $Q_{\textrm{tot}}(\tau , u, s)$, while enforcing a monotonic relationship between individual agent value functions and the joint value function, thereby enabling efficient decentralized decision-making.

QMIX maintains an individual DRQN for each agent to approximate its local action-value function $Q_i(\tau _i, u_i)$, where $\tau _t^i$ denotes the agent’s action–observation history. A mixing network then combines individual Q-values into a joint action-value function $Q_{\textrm{tot}}$ conditioned on the global state $s_t$, while satisfying the following monotonicity constraint:

3 $$\begin{aligned} \frac{\partial Q_{\textrm{tot}}}{\partial Q_i} \ge 0, \quad \forall i \in N \end{aligned}$$

This constraint guarantees that maximizing the joint action-value function can be achieved by independently maximizing each agent’s local Q-function, thus enabling decentralized execution. During training, QMIX minimizes the temporal-difference (TD) loss:

4 $$\begin{aligned} \mathcal {L}(\theta ) = \sum _{b=1}^{B} \left( Q_{\textrm{tot}}(\tau , u, s; \theta ) - \left( r + \gamma \max _{u'} Q_{\textrm{tot}}(\tau ', u', s'; \theta ^-) \right) \right) ^2 \end{aligned}$$

where *B* is the batch size, *θ* and $\theta ^-$ denote the parameters of the online and target networks, respectively, and *r* represents the total reward. This learning paradigm allows agents to estimate the long-term value of state–action pairs under joint strategies, which naturally aligns with the long-horizon strategic guidance provided by the LLM Commander.

#### Dynamic action masking

Although the LLM Commander operates in the symbolic and semantic space to generate high-level strategies, the low-level MARL agents still require numerical feature representations and must select concrete actions from discrete local action sets under QMIX decentralized execution. The dynamic action masking mechanism serves as an interface in LEHCA, translating LLM-generated task objectives and action constraints into numerical signals that can be combined with each agent’s utility $Q_i$ at decision time.

The sub-task objectives and action constraints produced by the LLM (e.g., “prioritize focus fire on enemy damage dealers” or “move south”) are parsed and converted into agent-specific hard masks and soft preference weights. The mask parsing module converts the LLM’s structured JSON output into per-agent tensors over $\mathcal {U}_i$: a hard mask $M_{\textrm{hard},i}(u)\in \{0,1\}$ marks actions that must not be executed under the current Commander guidance, and a soft weight $W_{\textrm{soft},i}(u)\in (0,\infty )$ encodes relative preferences among actions that remain feasible. Hard constraints enforce prohibitions, while soft constraints provide additive shaping of action scores rather than a standalone reparametrized distribution over actions.

#### Policy integration with dynamic masks

Within the QMIX decentralized execution interface, we integrate masks through *masked scores* $\tilde{Q}_i$ built from each agent’s DRQN utility $Q_i$ and the parsed masks, rather than by elementwise multiplication $Q_i\odot M_{\textrm{hard},i}\odot W_{\textrm{soft},i}$. Such products are avoided because zeroing disallowed entries does not reliably discard negative-valued actions under $\arg \max$, and because unstructured positive rescaling of raw *Q* can be numerically brittle relative to the scale of utilities. We write $\tau _i^t$ for agent *i*’s action–observation history at *t* (the same object as $\tau _t^i$ in the QMIX notation above). For each $u\in \mathcal {U}_i$, define

5 $$\begin{aligned} \tilde{Q}_i(\tau _i^t,u)= {\left\{ \begin{array}{ll} Q_i(\tau _i^t,u)+\beta \,\log W_{\textrm{soft},i}(u), & M_{\textrm{hard},i}(u)=1,\\ -\infty , & M_{\textrm{hard},i}(u)=0, \end{array}\right. } \end{aligned}$$

where $Q_i(\tau _i^t,u)$ is the DRQN utility output, $\beta \ge 0$ is a fixed coefficient controlling the strength of soft tilting ($\beta {=}0$ yields hard masking only), and $\log W_{\textrm{soft},i}$ is well-defined because $W_{\textrm{soft},i}(u)>0$ on $\mathcal {U}_i$. Decentralized greedy selection is

6 $$\begin{aligned} u_i^t = \arg \max _{u\in \mathcal {U}_i} \tilde{Q}_i(\tau _i^t,u). \end{aligned}$$

Because disallowed actions receive *-∞*, any maximizer of (6) lies in $\mathcal {U}_i^{\textrm{allowed}}=\{u\in \mathcal {U}_i: M_{\textrm{hard},i}(u)=1\}$ whenever $\mathcal {U}_i^{\textrm{allowed}}\ne \emptyset$. Under the same non-emptiness assumption, *\varepsilon*-greedy behavior uses: with probability *1-\varepsilon*, follow (6); with probability *\varepsilon*, draw uniformly from $\mathcal {U}_i^{\textrm{allowed}}$ (the exploratory branch never samples outside the allowed set). Ties in (6) may be broken arbitrarily. This procedure is a *practical integration* of Commander outputs with QMIX-style utility maximization; we do not assert theoretical optimality for the induced behavior policy relative to unconstrained team-optimal control or general constrained RL.

The same $Q_i(\tau _i^t,u)$ evaluated by the utility networks continues to feed the QMIX mixing network and TD backups on stored transitions; only the behavior policy that fills the replay buffer is obtained from (5)–(6).

Importantly, the action masks are dynamically updated rather than fixed. The LLM Commander continuously adjusts sub-task objectives, reward rules, and action constraints according to real-time environmental changes and evolving macro-strategies. The update process is formalized as:

7 $$\begin{aligned} M_{\textrm{hard},t+1}, W_{\textrm{soft},t+1} = \textrm{Update}(M_{\textrm{hard},t}, W_{\textrm{soft},t}, d_t, u_t, \pi _{\textrm{LLM}}^t) \end{aligned}$$

where $\textrm{Update}(\cdot )$ parses refreshed mask tensors from the Commander’s latest structured output $\pi _{\textrm{LLM}}^t$, grounded on the current semantic bundle $d_t$ and—when mask rules reference recent interaction—the joint action $u_t$. This bookkeeping keeps tactical learning aligned with the evolving strategic intent expressed in language, without implying that the LLM observes privileged simulator state beyond $d_t$.

### Computational complexity and deployment considerations

Relative to plain QMIX, LEHCA introduces additional computation along four conceptual channels: semantic state construction, intermittent Commander (LLM) inference, semantic reward evaluation, and dynamic action-mask generation.

Let *N* denote the number of agents, $|\mathcal {U}|$ the average local action-space size, *K* the number of active sub-goals, and $F_{\textrm{update}}$ the Commander refresh interval in environment steps. The per-step workload of the tactical stack remains dominated by the agent utilities and the mixing network, i.e., of the same asymptotic order as standard QMIX. Beyond that baseline, mask construction adds $O(N|\mathcal {U}|)$ work per step when masks are regenerated from the latest Commander output, while semantic shaping adds *O*(*K*) team-level checks and up to $O(K{+}N)$ when predicates are agent-specific. *Qualitatively*, the dominant additional expense is intermittent Commander inference: each refresh pays a large constant-factor LLM cost that plain QMIX does not incur. Under the fixed-refresh schedule used in this paper, one such call arises every $F_{\textrm{update}}$ steps, so a simple amortized bookkeeping expression for extra work per environment step is

8 $$\begin{aligned} C_{\textrm{extra}} \approx \frac{C_{\textrm{LLM}}(L)}{F_{\textrm{update}}} + O(N|\mathcal {U}| + K), \end{aligned}$$

where $C_{\textrm{LLM}}(L)$ denotes an abstract placeholder for the cost of one Commander call at prompt length *L*. Equation (8) is intended only as scaling intuition; we neither calibrate nor validate $C_{\textrm{LLM}}(L)$ with runtime measurements in this manuscript.

As tasks grow richer, qualitative pressure on overhead increases along three axes: longer textual summaries (more entities and relations), smaller $F_{\textrm{update}}$ (more frequent Commander calls), and larger *K* (more predicates to evaluate). Template-based compression, bounded JSON outputs, and choosing $F_{\textrm{update}}$ conservatively relative to the MARL step rate are standard mitigations; event-triggered refreshes could further cap calls.

From a deployment perspective, three practical points follow: a smaller or quantized Commander can reduce per-refresh expense when hardware is limited; parsed strategic outputs can be reused between refreshes to avoid redundant LLM calls; and the tactical agents can fall back to the last valid sub-goals if the Commander is temporarily unavailable, improving robustness at the expense of stale guidance.

Algorithm 1 summarizes the complete training procedure of LEHCA, encompassing LLM strategic updates, semantic reward shaping, dynamic action mask injection, and QMIX parameter optimization.

**Algorithm 1:**
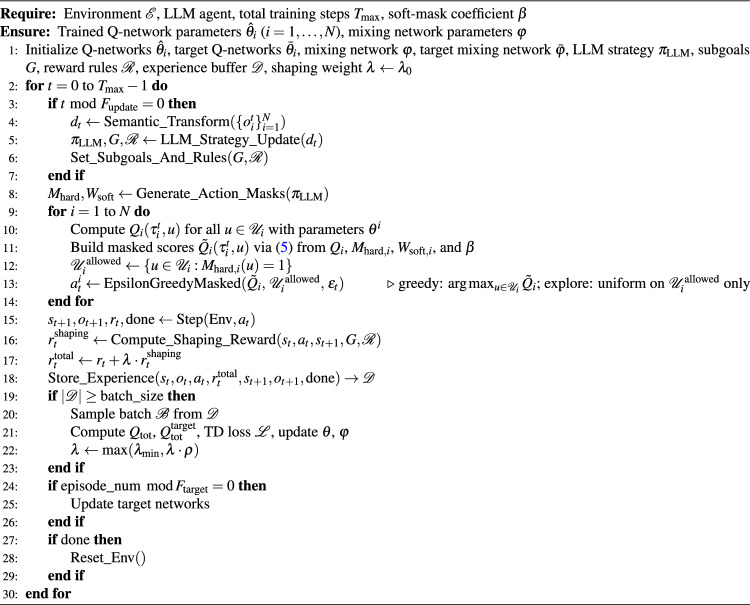
Training procedure of the LEHCA framework

## Experiments

This section reports empirical results for the proposed LLM-empowered hierarchical cognitive architecture (LEHCA) on SMAC and supporting analyses of training behavior and interpretability in the evaluated multi-agent settings.

### Experimental environment and benchmark tasks

#### SMAC environment

The experiments adopt the StarCraft multi-agent challenge (SMAC) as the primary evaluation platform. SMAC is one of the most representative and challenging benchmarks in multi-agent reinforcement learning, built upon the StarCraft II game engine and focusing on micromanagement combat tasks.

In the StarCraft multi-agent challenge (SMAC), cooperative decision-making necessitates that multiple agents operate under conditions of partial observability, within highly adversarial and non-stationary environments, to collectively optimize team-level performance. This domain is fundamentally characterized by decentralized execution, where agents rely solely on local observations; a centralized global reward signal provided by the environment; strong strategic coupling, meaning individual actions have significant, non-linear impacts on global outcomes; and intrinsic reward sparsity, as critical win/loss signals are typically only provided as episodic signals. These defining properties collectively establish SMAC as a rigorous and suitable testbed for evaluating hierarchical and strategy-guided multi-agent reinforcement learning frameworks such as LEHCA.

#### Scenario selection and complexity coverage

To evaluate LEHCA across different levels of task complexity and cooperation patterns, we select eight representative scenarios from the official SMAC benchmark. These scenarios cover homogeneous and heterogeneous teams, symmetric and asymmetric settings, as well as large-scale coordination challenges. The detailed configurations are summarized in Table 1.

**Table 1:** Representative SMAC scenarios.

Scenario type	Map	Friendly units	Enemy units	Core challenge
Homogeneous, symmetric	3m	3 Marines	3 Marines	Basic cooperation and focus fire
	8m	8 Marines	8 Marines	Large-scale coordination
Heterogeneous, symmetric	MMM	1 Medivac, 2 Marauders, 7 Marines	Same	Heterogeneous unit coordination
	2s3z	2 Stalkers, 3 Zealots	Same	Melee–ranged tactical synergy
Heterogeneous, asymmetric	2m_vs_1z	2 Marines	1 Zealot	Basic micromanagement
	3s_vs_5z	3 Stalkers	5 Zealots	High-difficulty combat
Outnumbered	5m_vs_6m	5 Marines	6 Marines	Numerical disadvantage
	27m_vs_30m	27 Marines	30 Marines	Large-scale coordination under pressure

These scenarios differ substantially in reward sparsity, number of agents, unit heterogeneity, and tactical complexity, yielding a varied testbed for assessing coordination difficulty and learning dynamics across the included maps.

#### Baselines and experimental settings

To provide a stronger yet practically feasible evaluation, the revised experiments compare LEHCA against baselines from multiple methodological families. Specifically, we include QMIX as the primary underlying value-factorization baseline, QPLEX as a stronger value-factorization method, and MAVEN as an exploration-oriented value-based MARL baseline. In addition, we report MAPPO on representative scenarios as a strong actor–critic baseline.

To isolate the contribution of the LLM itself while keeping the experimental design consistent with the QMIX-based architecture of LEHCA, we additionally construct three controlled variants that preserve the same low-level QMIX backbone and the same strategic update frequency while replacing the LLM Commander with non-LLM alternatives: (i) *Rule-Commander + QMIX*, in which sub-goals and masks are generated from handcrafted tactical heuristics; (ii) *Heuristic-Shaping + QMIX*, in which dense shaping rewards are manually designed based on domain priors; and (iii) *GRU-Commander + QMIX*, in which a compact learned recurrent controller replaces the LLM and outputs sub-goals at the same temporal resolution. These controls allow us to distinguish the contribution of semantic reasoning from the effect of hierarchy or auxiliary shaping alone.

All methods are trained under the same environment budget, evaluation protocol, random seeds, and hardware configuration. For the controlled variants, we use the same semantic state interface, sub-goal dimensionality, and Commander refresh interval $F_{\textrm{update}}$ as in the full LEHCA model to ensure a fair comparison. The LLM Commander and its non-LLM counterparts are invoked on this fixed schedule in all reported runs. To further examine cross-environment robustness with limited additional experimental cost, we also evaluate LEHCA and representative baselines on a simple cooperative task in the multi-agent particle environment (MPE).

Shared QMIX training hyperparameters, the Adam optimizer choice, and the LLM decoding defaults for the Commander are listed in Table 2. Table 3 then summarizes the extended baseline families and LLM-isolation controls used in the revised protocol.

**Table 2:** Common QMIX hyperparameter settings.

Setting	Value	Setting	Value
Learning rate	0.001	Optimizer	Adam
Batch size	128	LLM backbone	gpt-oss:20b
Discount factor *γ*	0.99	LLM temperature	0.2
Replay buffer size	5000	max_tokens	3072

**Table 3:** Extended baselines and controlled variants evaluated in the revised experiments.

Category	Method	Purpose
Value factorization	QMIX, QPLEX	Strong off-policy CTDE baselines
Exploration-oriented	MAVEN	Tests whether gains stem mainly from exploration
Actor–critic	MAPPO	Strong cross-family CTDE baseline
LLM-isolation control	Rule-Commander + QMIX	Handcrafted sub-goals and masks
LLM-isolation control	Heuristic-Shaping + QMIX	Manual dense reward baseline
LLM-isolation control	GRU-Commander + QMIX	Learned high-level controller without LLM

#### Evaluation metrics

To assess the performance of different methods in complex multi-agent environments, we evaluate algorithms along three complementary dimensions: final performance, sample efficiency (early-stage learning), and training stability.

*Win rate* is defined as the proportion of test episodes in which the agent team defeats the built-in enemy AI. It serves as the primary indicator of final cooperative performance and task success. Learning curves plot win rate as a function of training steps to analyze convergence speed and stability.

Final win rate alone may obscure differences in early-stage learning efficiency. To quantify how quickly effective behaviors emerge, we introduce the early-stage area under the learning curve, denoted as $\textrm{AUC}_{\textrm{early}}$. Let *y*(*t*) denote the average win rate at training step *t*. $\textrm{AUC}_{\textrm{early}}$ is defined as

9 $$\begin{aligned} \textrm{AUC}_{\textrm{early}} = \int _0^{T_{\textrm{early}}} y(t) \, dt \end{aligned}$$

where $T_{\textrm{early}}$ corresponds to the first 20% of total training steps. Since win rates are recorded at discrete time points, numerical integration is approximated using the trapezoidal rule. To ensure comparability across different scenarios, all $\textrm{AUC}_{\textrm{early}}$ values are normalized by $T_{\textrm{early}}$, resulting in values within [0, 1] that directly reflect the average early-stage task success rate. All tabulated results report the mean over five independent runs, and values are rounded to four decimal places for readability. Learning curves are also averaged over five random seeds to provide a stable estimate of performance and training stability.

### Training performance across different maps

**Fig. 2:**
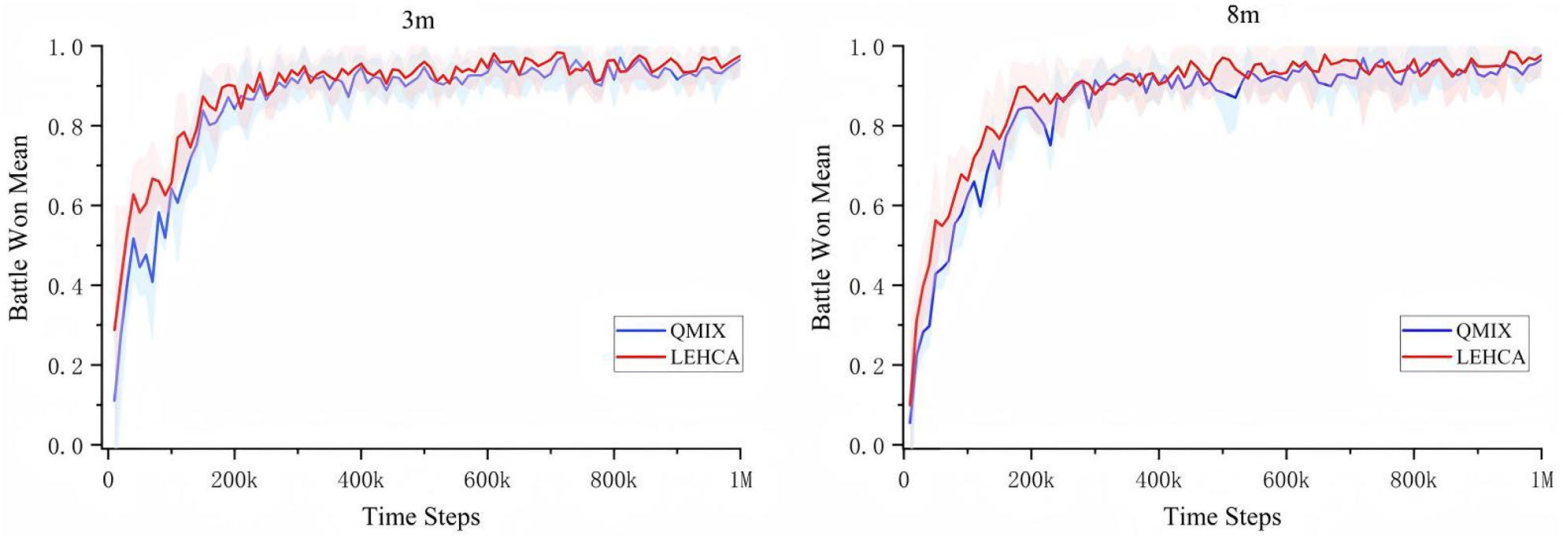
Win-rate comparison in homogeneous and symmetric scenarios.

**Fig. 3:**
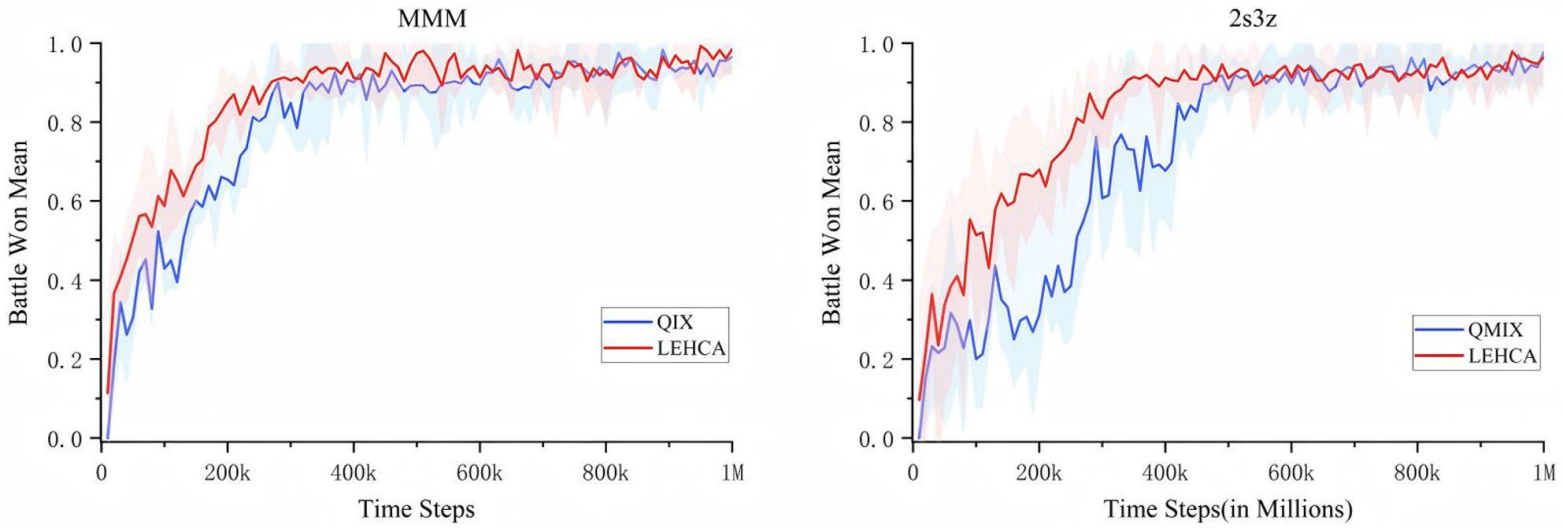
Win-rate comparison in heterogeneous and symmetric scenarios.

**Fig. 4:**
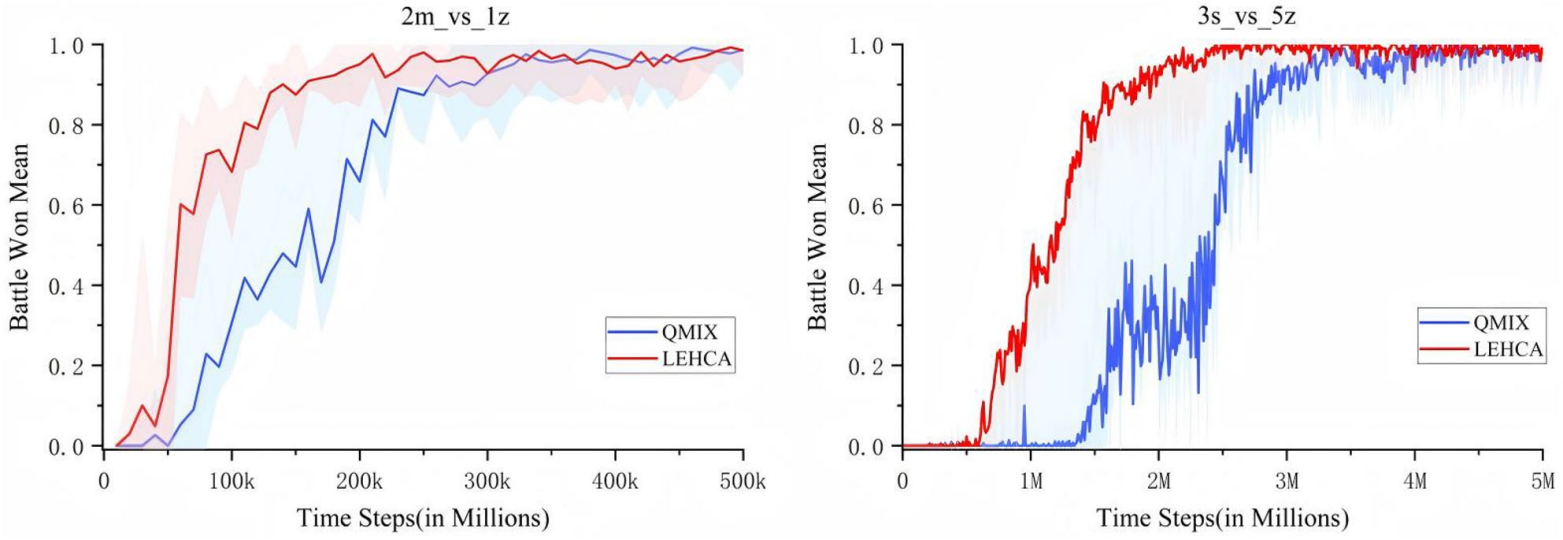
Win-rate comparison in heterogeneous and asymmetric scenarios.

**Fig. 5:**
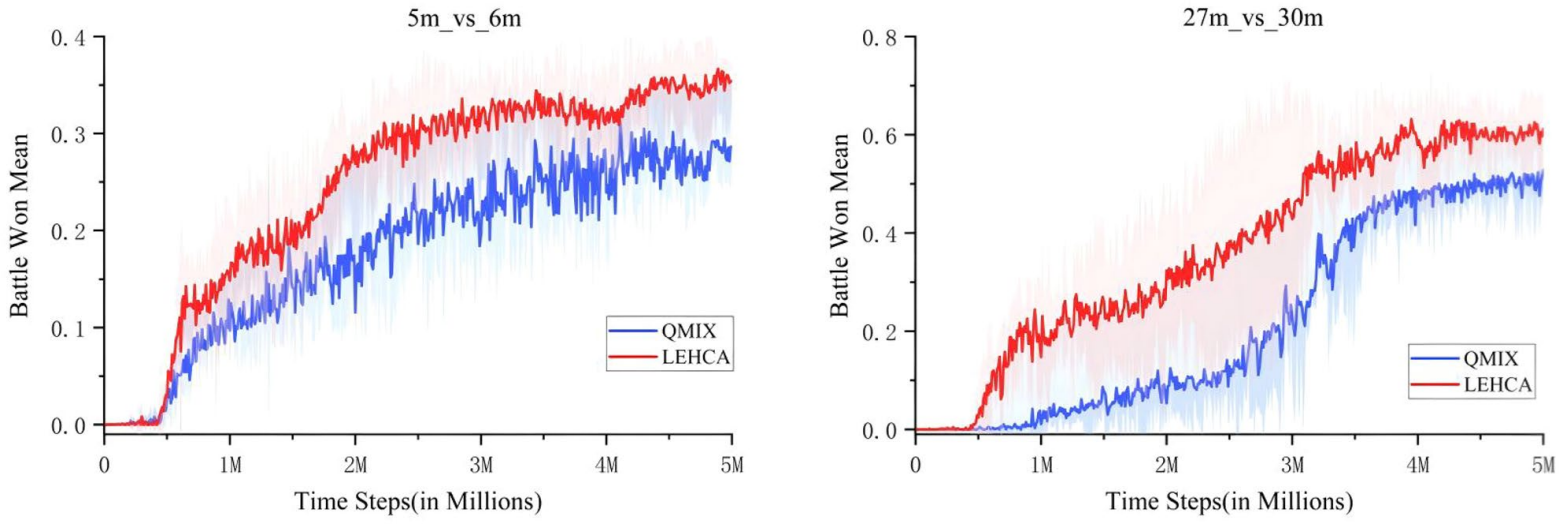
Win-rate comparison in outnumbered scenarios.

This section evaluates the proposed LLM-enhanced hierarchical cognitive architecture (LEHCA) across eight representative SMAC scenarios, covering homogeneous and heterogeneous teams, symmetric and asymmetric settings, as well as large-scale cooperative combat. As illustrated in Figs. 2, 3, 4 and 5, LEHCA improves over the QMIX baseline across the evaluated maps in our reported metrics, with comparatively larger margins in learning efficiency (including $\textrm{AUC}_{\textrm{early}}$), training stability, and final win rate on several harder coordination settings; the relative gaps tend to widen as task complexity and coordination difficulty increase, while differences can be modest where performance is already near saturation.

In relatively simple homogeneous scenarios (3m and 8m), both methods eventually converge to high win rates. However, LEHCA exhibits a faster rise in win rate early in training and noticeably lower variance throughout training in our runs. This indicates that even in environments with comparatively denser feedback, high-level strategic guidance and semantic reward shaping can effectively reduce redundant exploration and stabilize value learning. In the 8m scenario, QMIX exhibits a noticeable mid-training performance degradation, which is commonly associated with increased non-stationarity induced by a larger number of agents. In contrast, LEHCA maintains a smooth and largely monotonic improvement trajectory, highlighting its robustness to coordination-induced non-stationarity as the team size increases.

The advantages of LEHCA become more evident in heterogeneous symmetric scenarios (MMM and 2s3z), where agents with different roles and capabilities must coordinate precisely. QMIX exhibits pronounced oscillations and delayed stabilization, particularly in the 2s3z scenario, reflecting the increased difficulty of credit assignment and value estimation under heterogeneous interactions. By comparison, LEHCA rapidly achieves high and stable win rates. One plausible explanation is that the LLM-driven high-level task decomposition mechanism provides role-aware coordination cues and guides low-level agents toward strategically coherent behaviors. Leveraging the LLM’s semantic reasoning capability, LEHCA is able to form stable cooperation patterns—such as frontline tanking, backline damage dealing, and medical unit protection—at an earlier stage of training, resulting in substantially improved mid-training performance.

Asymmetric and sparse-reward scenarios (2m_vs_1z and 3s_vs_5z) further highlight the importance of long-horizon reasoning and structured exploration. Although both methods eventually achieve near-optimal performance in the simpler 2m_vs_1z scenario, LEHCA requires significantly fewer training steps to do so. In the considerably more challenging 3s_vs_5z scenario, QMIX remains trapped at near-zero win rates during early training and exhibits highly unstable learning dynamics. In contrast, LEHCA shows a clear phase transition in learning behavior: once high-level sub-goals begin to be consistently achieved, win rates increase rapidly and converge smoothly. This pattern is consistent with LEHCA mitigating, at least in part, long-term credit assignment difficulties by transforming sparse terminal rewards into structured intermediate learning signals.

The most challenging scenarios, 5m_vs_6m and 27m_vs_30m, are characterized by numerical disadvantage and large-scale coordination requirements. Experimental results show that QMIX learns slowly and achieves limited final performance in these settings, suggesting that monotonic value decomposition alone is insufficient for discovering effective global strategies. In contrast, LEHCA attains higher final win rates on these maps in our experiments and exhibits earlier performance improvements. Notably, the gap between LEHCA and QMIX tends to widen as the number of agents increases, which is consistent with comparatively more favorable scaling of the hierarchical design in these outnumbered settings, without implying optimality beyond the evaluated suite.

Overall, these results suggest that the proposed hierarchical cognitive decoupling can track increasing coordination difficulty more smoothly than QMIX in several of the reported settings and mitigates some of the pronounced performance degradation observed for QMIX on the hardest maps in our runs. These results suggest that the observed gains are not explained solely by additional architectural components, but are consistent with the intended division of cognitive responsibilities between high-level symbolic guidance and low-level reactive control. As task difficulty, heterogeneity, and agent population size increase, the benefits of this hierarchical division tend to become more prominent in our runs. In particular, the improvements are largest in scenarios characterized by sparse rewards, long-term dependencies, and high coordination demands among those we evaluate.

### Comparison with stronger baselines

To further evaluate LEHCA beyond the QMIX baseline, we compare it with three representative MARL methods on selected SMAC scenarios: QPLEX, MAVEN, and MAPPO. QPLEX is included as a stronger value-factorization baseline, MAVEN is used to assess the effect of exploration-oriented value-based learning, and MAPPO provides a competitive actor–critic reference. The comparison is conducted on four scenarios, 8m, 2s3z, 2m_vs_1z, and 5m_vs_6m, which cover homogeneous coordination, heterogeneous coordination, sparse-reward learning, and outnumbered combat.

**Table 4:** Comparison with stronger baselines on representative SMAC scenarios.

Method	8m		2s3z		2m_vs_1z		5m_vs_6m	
	Win Rate	AUC$_{\textrm{early}}$	Win Rate	AUC$_{\textrm{early}}$	Win Rate	AUC$_{\textrm{early}}$	Win Rate	AUC$_{\textrm{early}}$
QMIX	0.9673	0.5489	0.9733	0.2536	0.9920	0.0750	0.2950	0.0387
QPLEX	0.9758	0.5734	0.9709	0.3187	0.9934	0.0882	0.3385	0.0458
MAVEN	0.9739	0.5565	0.9814	0.4379	0.9881	0.2096	0.3496	0.0524
MAPPO	0.9616	0.5412	0.9748	0.3024	0.9863	0.1519	0.3117	0.0462
LEHCA	0.9867	0.6280	0.9785	0.4549	0.9925	0.3337	0.3672	0.0612

Table 4 reports the final win rate and early-stage learning performance of each method. On 8m, LEHCA achieves both the highest final win rate and the highest $\textrm{AUC}_{\textrm{early}}$, indicating that the proposed guidance mechanism improves learning speed even in a relatively saturated homogeneous setting. On 2s3z, MAVEN obtains a slightly higher final win rate than LEHCA, while LEHCA achieves the best $\textrm{AUC}_{\textrm{early}}$. This suggests that exploration-oriented methods remain highly competitive in heterogeneous combat, but the semantic guidance in LEHCA is more effective in accelerating the early formation of coordinated behaviors.

A similar pattern can be observed on 2m_vs_1z. QPLEX reaches the highest final win rate, although the difference from LEHCA is marginal. In contrast, LEHCA substantially improves $\textrm{AUC}_{\textrm{early}}$ compared with all baselines, showing that it reaches effective policies earlier during training. This distinction is important because final performance on this scenario is close to saturation for several methods, making early-stage learning efficiency a more informative metric for comparing training dynamics.

The advantage of LEHCA is most evident on 5m_vs_6m, where agents must coordinate under numerical disadvantage. In this scenario, LEHCA achieves the highest final win rate and the highest $\textrm{AUC}_{\textrm{early}}$, outperforming QMIX, QPLEX, MAVEN, and MAPPO. These results indicate that high-level semantic guidance is particularly beneficial when the task requires coordinated target selection, role allocation, and sustained strategic consistency over a longer horizon.

Overall, the results in Table 4 show that LEHCA is not simply benefiting from a stronger value-factorization backbone or from enhanced exploration alone. While QPLEX and MAVEN remain competitive in some final win-rate comparisons, LEHCA achieves the best early-stage learning performance across all four scenarios and delivers the strongest results on the most coordination-demanding map. These findings support the effectiveness of combining LLM-based high-level guidance with low-level MARL optimization, especially for improving sample efficiency and stabilizing coordination in challenging cooperative tasks.

### Ablation study

To precisely quantify the contribution of each core component in LEHCA, we conduct a systematic ablation study focusing on the effects of semantic reward shaping and dynamic action masking across scenarios with different levels of complexity. The evaluation metrics include the final average win rate and the early learning area under the curve $\textrm{AUC}_{\textrm{early}}$, where $\textrm{AUC}_{\textrm{early}}$ characterizes the efficiency with which effective cooperative strategies are acquired during the early training stage.

We constructed two key ablation variants: the first, LEHCA without dynamic reward shaping, retains the LLM’s generation of high-level strategies and sub-goals but removes the provision of dense semantic reward signals, thereby training the MARL agents solely on sparse environmental rewards; the second, LEHCA without dynamic action masking, maintains the LLM’s strategic guidance, sub-goal generation, and reward shaping but disables the injection of its action constraints via the dynamic masking mechanism, allowing agents to explore the full action space without explicit strategic pruning.

These settings are designed to separately evaluate the role of reward shaping in alleviating sparse-reward challenges and accelerating exploration, and the role of action masking in maintaining consistency between high-level strategies and low-level execution. Table 5 reports the ablation results on four representative SMAC scenarios (8m, 2s3z, 2m_vs_1z, and 5m_vs_6m), covering homogeneous cooperation, heterogeneous coordination, sparse-reward settings, and highly challenging outnumbered scenarios.

**Table 5:** Ablation results. Reward: semantic reward shaping; mask: dynamic action masking.

Reward	Mask	8m		2s3z		2m_vs_1z		5m_vs_6m	
		Win Rate	AUC$_{\textrm{early}}$	Win Rate	AUC$_{\textrm{early}}$	Win Rate	AUC$_{\textrm{early}}$	Win Rate	AUC$_{\textrm{early}}$
*×*	*×*	0.9673	0.5489	0.9733	0.2536	0.9920	0.0750	0.2950	0.0387
*√*	*×*	0.9755	0.5923	0.9753	0.3425	0.9914	0.2126	0.3246	0.0505
*×*	*√*	0.9721	0.5654	0.9740	0.3100	0.9911	0.1349	0.3154	0.0446
*√*	*√*	0.9867	0.6280	0.9785	0.4549	0.9925	0.3337	0.3672	0.0612

As shown in Table 5, removing semantic reward shaping leads to a substantial degradation in $\textrm{AUC}_{\textrm{early}}$ across all scenarios, with the effect being particularly pronounced in sparse-reward and coordination-intensive settings. For example, in the 2s3z and 2m_vs_1z scenarios, models trained solely with environment rewards struggle to form effective cooperative behaviors during early training, resulting in $\textrm{AUC}_{\textrm{early}}$ values decreasing from 0.4549 and 0.3337 in the full model to 0.3425 and 0.2126, respectively. These results indicate that semantic reward shaping effectively translates high-level strategic intent into structured intermediate learning signals, thereby mitigating long-term credit assignment issues and exploration difficulties induced by sparse rewards.

In contrast, removing dynamic action masking yields a more moderate but systematic performance drop. While learning efficiency is partially preserved, both $\textrm{AUC}_{\textrm{early}}$ and final win rate remain consistently lower than those of the complete model, with the performance gap widening as task difficulty increases. In the challenging 5m_vs_6m scenario, this ablation variant achieves a final win rate of 0.3154 and an $\textrm{AUC}_{\textrm{early}}$ of 0.0446, compared to 0.3672 and 0.0612 achieved by the full LEHCA model. This suggests that reward signals alone are insufficient to guarantee alignment between high-level strategic intent and low-level policy search in the absence of explicit action constraints, leading to inefficient exploration and coordination mismatches.

It is worth noting that in relatively simple scenarios such as 8m, where performance is close to saturation, differences in final win rate among ablation variants are relatively small. However, $\textrm{AUC}_{\textrm{early}}$ continues to clearly differentiate learning efficiency across configurations. This observation further validates the necessity of $\textrm{AUC}_{\textrm{early}}$ as a complementary metric, as relying solely on final performance may obscure meaningful differences in learning dynamics.

Overall, the complete LEHCA configuration achieves the highest reported final win rates and $\textrm{AUC}_{\textrm{early}}$ values on the ablation subset in Table 5. These results are consistent with a synergistic interaction between semantic reward shaping and dynamic action masking under the tested protocol. The former provides directionally informative, high-level guidance for the multi-agent learning process, while the latter effectively constrains policy search within strategically meaningful regions of the action space. Together, they enable more efficient, stable, and scalable cooperative learning.

### LLM contribution isolation

The preceding ablation study examines the roles of semantic reward shaping and dynamic action masking, but these results do not by themselves separate the effect of the LLM Commander from the broader hierarchical design. To further analyze this issue, we construct three controlled variants that keep the same QMIX low-level learner, semantic state interface, and strategic update interval as LEHCA, while replacing the LLM Commander with alternative high-level guidance mechanisms.

The first variant, Rule-Commander + QMIX, uses handcrafted tactical rules to generate sub-goals and action constraints from the same semantic state description. The second variant, Heuristic-Shaping + QMIX, removes LLM-generated reward rules and instead uses manually designed dense rewards based on common combat heuristics, such as target elimination and ally protection. The third variant, GRU-Commander + QMIX, replaces the LLM with a compact recurrent controller that receives the same structured state input and outputs high-level sub-goals at the same temporal resolution. Since the low-level learner and update schedule are unchanged across these variants, the comparison focuses on the source and expressiveness of the high-level guidance.

Table 6 reports the results on 2s3z, 2m_vs_1z, and 5m_vs_6m. These scenarios were selected because they involve different forms of coordination difficulty, including heterogeneous unit cooperation, sparse early feedback, and outnumbered combat. Across the three maps, LEHCA obtains the highest final win rate and the highest $\textrm{AUC}_{\textrm{early}}$, indicating that replacing the LLM Commander with rule-based, heuristic, or learned recurrent guidance reduces both final performance and early-stage learning efficiency.

**Table 6:** Isolating the contribution of the LLM Commander. All methods use the same QMIX low-level backbone and the same strategic update interval.

Method	2s3z		2m_vs_1z		5m_vs_6m	
	Win rate	AUC$_{\textrm{early}}$	Win rate	AUC$_{\textrm{early}}$	Win rate	AUC$_{\textrm{early}}$
Rule-Commander + QMIX	0.9658	0.3921	0.9716	0.3017	0.3214	0.0583
Heuristic-Shaping + QMIX	0.9716	0.4015	0.9882	0.2284	0.3348	0.0446
GRU-Commander + QMIX	0.9742	0.3787	0.9639	0.2392	0.3465	0.0508
LEHCA	0.9785	0.4549	0.9925	0.3337	0.3672	0.0612

The comparison also shows that non-LLM guidance is not without value. Rule-Commander + QMIX achieves relatively strong $\textrm{AUC}_{\textrm{early}}$ on 2m_vs_1z and 5m_vs_6m, suggesting that manually designed tactical priors can accelerate learning when the task structure is simple or when the relevant combat rule is easy to specify. Heuristic-Shaping + QMIX performs competitively in final win rate on 2m_vs_1z, which is consistent with the fact that this scenario is comparatively simple and can benefit from dense handcrafted reward signals. GRU-Commander + QMIX also improves over purely low-level learning in some cases, showing that a learned high-level controller can capture part of the useful temporal abstraction.

Nevertheless, the full LEHCA model provides the most balanced performance across all three scenarios. The gap is especially visible in $\textrm{AUC}_{\textrm{early}}$ on 2s3z and 2m_vs_1z, where LLM-generated sub-goals and reward rules appear to provide more informative guidance during the early stages of training. On the harder 5m_vs_6m map, LEHCA also achieves the best final win rate, indicating that semantic guidance remains useful when agents must coordinate under numerical disadvantage.

These results suggest that the performance improvement of LEHCA is not solely due to adding hierarchy or auxiliary rewards. Handcrafted and learned non-LLM guidance can recover part of the benefit, but their effectiveness varies more strongly across scenarios. In contrast, the LLM Commander provides a unified mechanism for generating sub-goals, reward rules, and action constraints from semantic state descriptions, reducing the need for scenario-specific rule engineering while maintaining strong early learning and final performance across the tested settings.

### Generalization to a lightweight cooperative MPE task

To examine whether the proposed guidance mechanism can be applied outside SMAC micromanagement scenarios, we further evaluate LEHCA on the MPE *simple_spread* cooperative navigation task. In this environment, multiple agents must coordinate to cover distinct landmarks while avoiding collisions. Compared with SMAC, the task has simpler dynamics, a shorter effective horizon, and denser feedback through landmark-distance rewards and collision penalties.

The LEHCA pipeline is kept unchanged at the framework level. Geometric observations are first converted into structured natural-language summaries, from which the Commander generates high-level coordination guidance, such as landmark coverage priorities, collision-avoidance preferences, and assignments that reduce redundant movement toward the same target. The low-level policies are still trained with the QMIX-based learner, using the same form of semantic reward shaping and action-guidance interface as in the SMAC experiments.

**Fig. 6:**
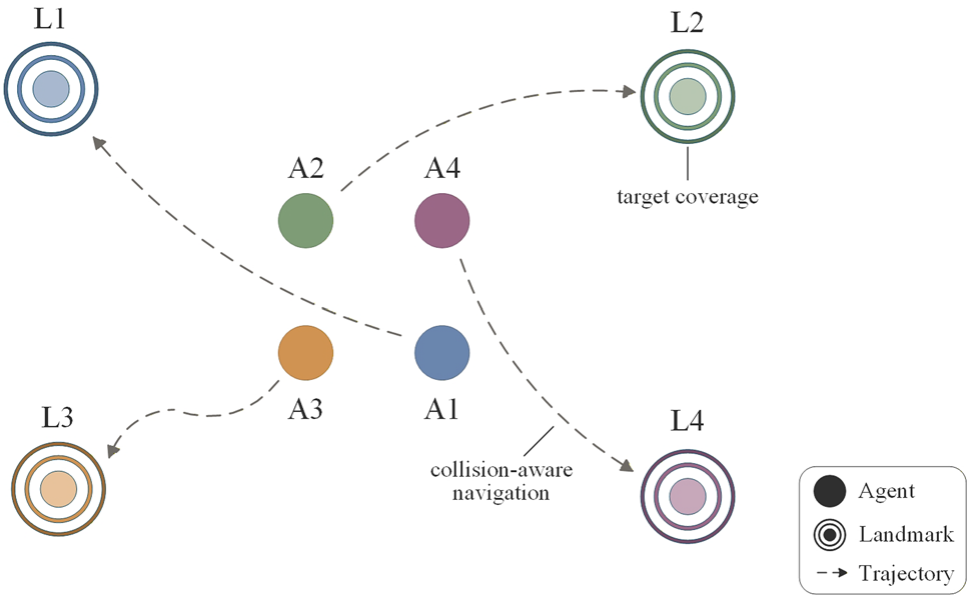
Illustration of the cooperative navigation task in the multi-agent particle environment (MPE).

For evaluation, *Final success rate* denotes the fraction of evaluation episodes in which the agent team successfully covers all landmarks under the same distance-based threshold for all compared methods. *Collision rate* denotes the normalized frequency of collision events during evaluation, where a lower value indicates smoother multi-agent coordination. Figure 6 illustrates the cooperative navigation setting used in this experiment.

As shown in Table 7, LEHCA achieves the highest final success rate and the highest $\textrm{AUC}_{\textrm{early}}$ among the compared methods, while also producing the lowest collision rate. Compared with QMIX, the improvement in $\textrm{AUC}_{\textrm{early}}$ indicates faster acquisition of effective landmark coverage behaviors, and the reduction in collision rate suggests that the high-level guidance helps agents distribute themselves more smoothly across landmarks. QPLEX also improves final success over QMIX, but its collision rate remains higher, indicating that stronger value factorization alone does not necessarily lead to better collision-aware coordination. MAVEN achieves a relatively low collision rate, but its final success rate is lower than that of LEHCA, suggesting that exploration-oriented behavior does not fully address the coverage objective in this setting.

The performance gap on MPE is smaller than the gains observed on the more difficult SMAC maps, because *simple_spread* already provides denser intermediate feedback and involves less complex tactical interaction. Nevertheless, the results show that the semantic guidance mechanism can be instantiated in a qualitatively different cooperative control task and can improve both task success and coordination quality. This experiment therefore provides supporting evidence that LEHCA is not tied exclusively to StarCraft combat dynamics, while broader validation on more diverse multi-agent domains remains a direction for future work.

**Table 7:** Results on a cooperative MPE task.

Method	Final success rate	AUC$_{\textrm{early}}$	Collision rate
QMIX	0.891	0.604	0.138
QPLEX	0.907	0.632	0.146
MAVEN	0.884	0.621	0.119
LEHCA	0.936	0.665	0.101

### Case study and interpretability analysis

To further illustrate the interpretability and effectiveness of the proposed LLM-empowered hierarchical cognitive architecture (LEHCA) in complex heterogeneous cooperation, we conduct a qualitative case study on the MMM scenario (1 Medivac, 2 Marauders, and 7 Marines vs. the same composition) from the StarCraft multi-agent challenge (SMAC). This scenario features balanced unit compositions and rich tactical dimensions, making it particularly suitable for analyzing coordinated decision-making and role differentiation.

**Fig. 7:**
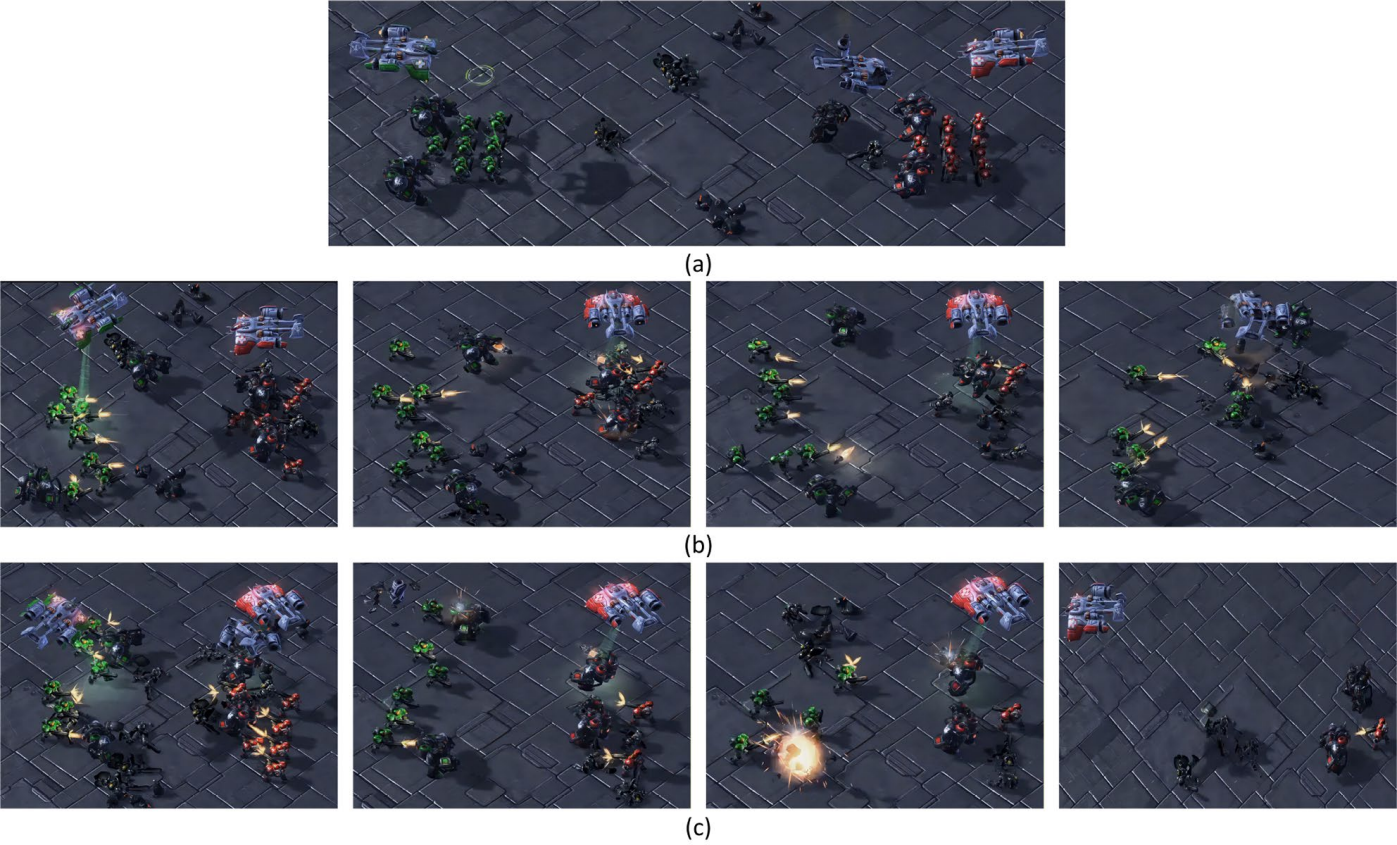
Engagement process in a typical scenario. (**a**) Initial state; (**b**) successful case; (**c**) failure case.

Building on the quantitative observations reported in the preceding subsections (including the MPE transfer experiment), we next provide a process-level interpretability analysis. Figure 7a shows the initial state of the MMM scenario, where both allied and enemy teams have identical formations. Allied units are shown in green, enemy units in red, and grey units represent casualties from previous episodes, which do not affect the current battle. Figure 7b presents a successful combat episode executed by LEHCA, with several key frames extracted. In this episode, the allied team eliminates all enemy units at the cost of losing only a single Medivac. In contrast, Fig. 7c illustrates a failed episode produced by a traditional MARL policy (QMIX baseline), where all allied units are eliminated while the enemy retains one Medivac, one Marauder, and one Marine.

In the successful LEHCA-controlled episode, the allied team maintains a coherent formation during the early phase of the battle. When the enemy focuses fire on the allied Medivac, the agents quickly adapt by prioritizing and focusing fire on enemy Marines, leading to a rapid reduction in the opponent’s damage-dealing units. Subsequently, allied Marauders take frontline positions to absorb damage while the remaining agents concentrate fire on enemy Marauders and surviving Marines. Finally, the allied team systematically eliminates the enemy Medivac, securing victory.

By contrast, in the failed baseline episode, Marines act largely independently, distributing their fire across multiple enemy unit types (Marauders, Marines, and Medivac) without forming an effective focus-fire pattern. This fragmented behavior prevents the team from neutralizing key threats and results in the allied units being eliminated sequentially.

LEHCA makes the decision-making process of MARL more interpretable by exposing a hierarchical guidance pipeline composed of LLM-generated rationale-style summaries and structured sub-goals. Through LLM-based semantic reward shaping, sparse terminal win/loss signals are converted into dense, semantically meaningful intermediate rewards, providing clear learning guidance for low-level agents. At the same time, the dynamic action masking mechanism offers fine-grained constraints during action selection, aligning low-level behaviors with high-level strategic intent.

**Fig. 8:**
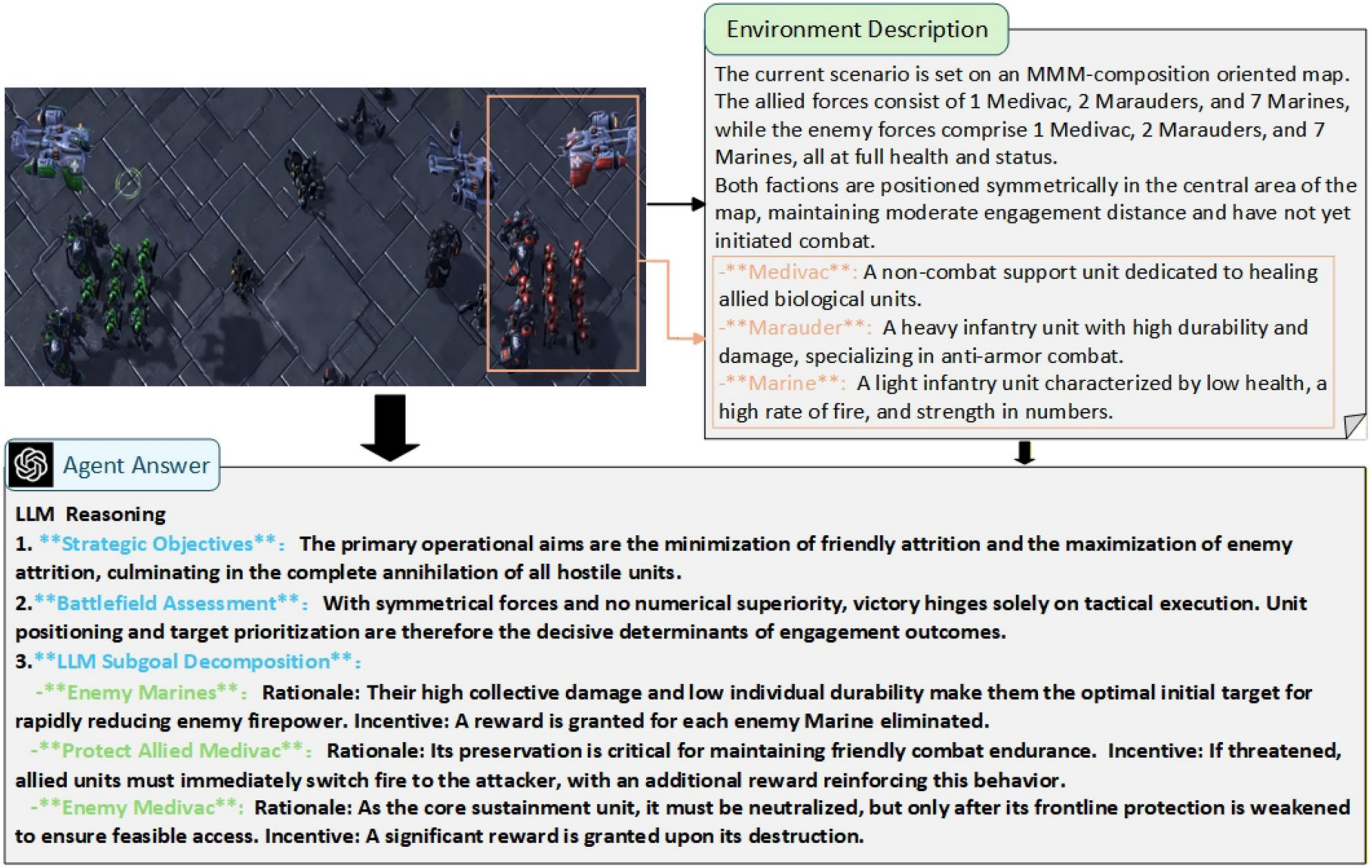
Description of the MMM combat scenario and the logical reasoning process of the LLM.

Figure 8 illustrates the structured natural language description of the MMM combat scenario and the corresponding reasoning process of the LLM. Based on the initial state description provided by the environment interaction module, the LLM performs semantic analysis of the battlefield, identifies priority threats, and formulates a set of sub-goals and reward shaping rules. These high-level decisions subsequently influence agent behavior through both reward modulation and action masking, resulting in coordinated and strategically coherent combat execution.

This case study suggests that LEHCA can improve quantitative performance in the illustrated episode while also yielding a comparatively transparent and human-interpretable decision-making trace. The observed behaviors—such as role-aware positioning, focus fire on high-impact targets, and adaptive response to enemy actions—closely align with human tactical reasoning in real-time strategy games. These observations suggest the practical value of integrating symbolic guidance and hierarchical cognition into multi-agent reinforcement learning, particularly in complex and heterogeneous cooperative environments.

## Conclusion

This paper presented LEHCA, a hierarchical multi-agent reinforcement learning framework that integrates large-language-model-based high-level guidance with value-decomposition-based low-level policy learning. The proposed framework separates strategic reasoning from tactical execution: the LLM Commander operates on structured textual summaries of observable environment information and generates sub-goals, reward-shaping rules, and action constraints, while the low-level agents learn executable policies through a QMIX-based MARL backbone. In this design, the underlying learner retains CTDE-style centralized training and decentralized tactical execution, whereas the overall system introduces an additional coarse-timescale guidance layer.

The main methodological contribution of LEHCA lies in connecting semantic guidance with numerical reinforcement learning through two mechanisms. First, LLM-driven semantic reward shaping maps abstract sub-goals into dense auxiliary learning signals, providing intermediate feedback in sparse-reward and long-horizon settings. Second, dynamic action masking translates high-level task constraints into action-level guidance, helping agents explore strategically relevant regions of the action space while preserving low-level policy learning within the MARL framework. These components are implemented as modular guidance mechanisms and do not require changing the core QMIX utility and mixing networks.

Experiments on eight SMAC scenarios show that LEHCA improves over QMIX across the evaluated maps in the reported metrics, with more visible gains in heterogeneous, sparse-reward, and outnumbered scenarios. Additional comparisons with QPLEX, MAVEN, and MAPPO on representative maps indicate that LEHCA is particularly effective in improving early-stage learning efficiency, as reflected by $\textrm{AUC}_{\textrm{early}}$, while maintaining competitive final performance. The ablation results further show that semantic reward shaping and dynamic action masking contribute complementary benefits. Controlled comparisons with Rule-Commander, Heuristic-Shaping, and GRU-Commander variants suggest that hierarchy and auxiliary guidance are useful in their own right, while the LLM Commander provides a more flexible source of high-level semantic guidance across the tested scenarios. The additional MPE cooperative navigation experiment further indicates that the framework can be instantiated beyond StarCraft micromanagement, although broader validation remains necessary.

The proposed framework also introduces limitations. LEHCA incurs additional computational overhead due to semantic state construction, intermittent LLM inference, reward grounding, and mask generation. Although the fixed Commander refresh interval amortizes part of this cost over multiple low-level environment steps, the framework should not be interpreted as improving wall-clock efficiency over QMIX. Its practical deployment may require smaller or quantized language models, cached high-level guidance, or adaptive invocation schedules. In addition, the quality of semantic reward shaping and action masking depends on the reliability of the semantic grounding rules and the executability of LLM-generated sub-goals.

Future work will extend LEHCA to more diverse multi-agent domains, including tasks with continuous control, mixed cooperation–competition, and more complex communication constraints. Another important direction is to develop adaptive Commander invocation strategies that balance guidance quality and computational cost. Finally, integrating more robust grounding and verification mechanisms for LLM-generated strategies may further improve the reliability of hierarchical LLM-guided MARL in practical multi-agent systems.

## Supplementary Information

Supplementary Information.

## Data Availability

The implementation of the proposed hierarchical multi-agent reinforcement learning framework, including the semantic reward shaping and task-guided policy learning modules, will be made publicly available upon publication of this article. All experiments were conducted using publicly available benchmark environments, including the StarCraft multi-agent challenge (SMAC). Detailed network architectures, hyperparameter settings, and training protocols are provided in the manuscript and supplementary materials.

## Declarations

### Use of artificial intelligence

The authors declare that large language models (LLMs) were used as an integral component of the proposed methodology, serving as a high-level cognitive and strategic reasoning module within the multi-agent reinforcement learning framework. The LLM was employed for situation understanding, long-term goal planning, task decomposition, and semantic reward shaping as part of the designed system. No generative AI tools were used for writing, editing, or generating the manuscript text.
