# Supplementary material for: A hierarchical multi-agent reinforcement learning framework with high-level guidance from large language models
Source: Sci Rep. 2026 May 25;16:23832. doi: 10.1038/s41598-026-54971-6 (PMC13434813; doi:10.1038/s41598-026-54971-6)
Supplement: Supplementary file 1 [file 41598_2026_54971_MOESM1_ESM.pdf]

An LLM-Empowered Hierarchical Cognitive Architecture for Interpretable and Scalable Multi-Agent Reinforcement Learning

This document provides additional methodological details that support the main manuscript. All experiments use the publicly available StarCraft Multi-Agent Challenge (SMAC) benchmark and the implementation is based on the PyMARL framework with LEHCA extensions.

1. Supplementary Methods

Environment: StarCraft II via the SMAC benchmark. Each scenario defines a map (e.g., `3m`, `5m_vs_6m`, `6h_vs_8z`) with a fixed number of allied and enemy units.

Observations: Per-agent local observation (e.g., unit features, ally/enemy visibility); optional global state for the mixer.

Actions: Discrete: no-op, move (4 directions), attack (4 directions + attack target for each enemy). Total action dimension is environment-dependent (e.g., 6 + n_enemies for attack actions).

Rewards: Sparse win/loss and optional death/kill rewards; reward scaling and shaping are as specified in the SMAC and SC2 env config (e.g., `reward_scale`, `reward_scale_rate`, `reward_win`, `reward_death_value`).

2. MARL Module (QMIX)

Agent network: `n_rnn` (recurrent agent). Input: observation (and optionally last action, agent id if enabled). Architecture: Linear(obs_dim, rnn_hidden_dim) → ReLU → GRUCell(rnn_hidden_dim, rnn_hidden_dim) → Linear(rnn_hidden_dim, n_actions). Output: Q-values per action.

Mixer: QMIX mixer with `mixing_embed_dim` and hypernet embedding dimension `hypernet_embed`.

Training: Standard QMIX loss (TD target, mixing); replay buffer of episodes; target network updated every `target_update_interval` steps.

3. Training Protocol and Hyperparameters

Env config: Map is set per run (e.g., `3m`, `5m_vs_6m`, `6h_vs_8z`).

mixing_embed_dim | 32 | Mixer embedding dimension |

hypernet_embed | 64 | Hypernet embedding dimension |

action_selector | epsilon_greedy | Epsilon-greedy action selection |

epsilon_start | 1.0 | Initial exploration rate |

epsilon_finish | 0.05 | Final exploration rate |

epsilon_anneal_time | 300000 | Steps for epsilon annealing |

rnn_hidden_dim | 64 | RNN hidden size (from default config) |
